# Will Medical Schools Train AI Tutors to Teach by Humiliation?

**DOI:** 10.1111/jep.70414

**Published:** 2026-03-09

**Authors:** Bilal Irfan, Roberto Daniel Sirvent

**Affiliations:** ^1^ Center for Bioethics Harvard Medical School Boston Massachusetts USA; ^2^ Center for Surgery and Public Health Brigham & Women's Hospital Boston Massachusetts USA; ^3^ Department of Neurology University of Michigan Medical School Ann Arbor Michigan USA; ^4^ Department of Epidemiology University of Michigan School of Public Health Ann Arbor Michigan USA; ^5^ Department of Global Health and Social Medicine Harvard Medical School Boston Massachusetts USA

**Keywords:** artificial intelligence in education, teaching by humiliation

The rapid integration of artificial intelligence into clinical and educational workflows invites a familiar warning from digital health: any system may only be as good as the data and practices that shape it. That axiom is usually applied to diagnostic algorithms and decision support, but it also belongs in the classroom. If educational models are trained on the habits, signals, and outcomes that dominate medical schooling, they will likely reproduce not only content but pedagogy. One entrenched practice in clinical training is teaching by humiliation, a pattern of public shaming, belittlement, and staged embarrassment used to compel performance [1]. When an approach is both prevalent and rationalized as effective, it may be legible to data collection and optimization routines as a norm to emulate. The result could be a generation of AI tutors that encode humiliation as a feature rather than a flaw.

Recent work on AI tutoring shows the promise and the peril. Investigators designed an AI tutor around what they considered pedagogical best practices, including active learning, cognitive load management, growth mindset, scaffolding, and timely feedback [2]. The intervention outperformed in‐class active learning. This is encouraging, yet it also raises a basic question for medical education: who decides what counts as a best practice, and what evidence base feeds that designation. Much of contemporary educational technology remains steeped in behaviorist assumptions that privilege extrinsic motivators, rigid outcome alignment, and compliance with summative assessment regimes. If training corpora and telemetry reward rapid fact retrieval, error avoidance, and test performance, AI tutors will optimize for those signals. They may neglect curiosity, conceptual integration, and clinical judgment under uncertainty, which are the very capacities that matter at the bedside.

Medical education's relationship with humiliation is well documented. This has included high rates of belittlement, public put‐downs, and disparaging responses to student questions [3, 4]. Qualitative studies show how yelling and derision have been normalized to the point that learners struggle to label them as mistreatment [5]. This culture is not reducible to loud voices. It also expresses itself through silence, facial cues of disdain, and the choreography of clinical rounds where a wrong answer becomes a spectacle. If AI tutors learn from this environment, they may not need to shout to humiliate. They can deploy leaderboards, streaks, and punitive hints that rank students against peers and convert error into social exposure [6].

Gamified platforms are often marketed as engines of engagement, but they frequently trade intrinsic motivation for points, badges, and hierarchical comparison. Leaderboards and competitive scoring introduce a persistent public record that quietly extends teaching by humiliation into the digital sphere. In parallel, the broader medical culture has struggled to create spaces where error reporting and uncertainty are not penalized [7]. When grades, scores, and public ranking dominate the reward structure, learners may predictably adapt to avoid embarrassment rather than to understand. An AI tutor that calibrates feedback to maximize time‐on‐task within this incentive ecology may inadvertently amplify the very harms educators claim to oppose.

The risks are not confined to software that interacts directly with students. Faculty can conscript AI as an authority during rounds. One can easily imagine a senior physician asking an assistant whether a trainee's provisional diagnosis is correct in front of a team, thereby outsourcing both adjudication and spectacle to a machine. Students, for their part, may turn to AI to survive humiliation rather than to learn. Cramming with a chatbot to avoid being singled out the next morning can boost short‐term performance while undermining retention. Evidence from basic science education suggests that large fractions of factual knowledge decay over one to 2 years, especially when learned for tests and rote recall rather than anchored in meaning and real understanding [8]. If AI becomes a shield against shame, it could reinforce extrinsic motivation and the forgetting that follows.

The path forward requires attention to both evidence and design. First, the field needs quantitative, qualitative, and mixed‐methods studies that examine the effects of humiliation on learning, mental health, and professional identity formation, and that test alternatives grounded in intrinsic motivation, problem‐solving, and cultivating a desire to explore new questions and ideas. Without such work, model developers may reach for whatever signals are most easily harvested, and humiliation's apparent efficacy in driving short‐run performance will be mistaken for pedagogy. Second, educators should stop using humiliation in any guise. Cultural practices that are rare are harder for data pipelines to learn, reproduce, and legitimate. Reducing the prevalence of humiliation will change the training distribution for both human and artificial teachers. Third, those building AI tutors for medical education must center on how teaching is conducted, not only what is taught. Systems should privilege formative over summative interaction, invite reflective error, scaffold conceptual understanding, and make progress visible to the learner without public comparison. Governance frameworks in digital health rightly emphasize safety, transparency, and bias; they should also scrutinize the motivational economies that AI introduces into classrooms and wards. If we fail to do so, the future of medical education may be more efficient but far less humane.

## Funding

The authors received no specific funding for this work.

## Disclosure

The authors have nothing to report.

## Conflicts of Interest

The authors declare no conflicts of interest.

## Data Availability

The data that support the findings of this study are available from the corresponding author upon reasonable request.
